# Development of a scale to assess motivation for competitive employment among persons with severe mental illness

**DOI:** 10.1371/journal.pone.0204809

**Published:** 2018-10-02

**Authors:** Natsuki Sasaki, Sayaka Sato, Sosei Yamaguchi, Michiyo Shimodaira, Norito Kawakami

**Affiliations:** 1 Department of Community Mental Health & Law, National Institute of Mental Health, National Center of Neurology and Psychiatry, Kodaira, Japan; 2 Department of Mental Health, Graduate School of Medicine, The University of Tokyo, Tokyo, Japan; Tokyo Metropolitan Institute of Medical Science, JAPAN

## Abstract

**Background:**

The employment rate among people with severe mental illness has recently increased, though it is still low. The motivation to work appears to be an important role as an intermediate outcome measure in vocational rehabilitation programs. In addition, measuring the work motivation for people with severe mental illness appears to be essential to identify candidates who are likely to benefit and monitor candidates’ motivation in a supported employment program. This study aimed to develop a new measure for assessing both intrinsic and extrinsic motivation to work among people with severe mental illness, as there are currently no well-established instruments of this kind.

**Methods:**

A focus group interview and review of previous qualitative research were used to identify possible items for inclusion in the new scale. A provisional scale was constructed and further refined for content and format based on feedback from a researcher and also three peer workers with severe mental illness. The resulting provisional 38-item version of the scale was completed by 136 respondents with severe mental illness, and we performed exploratory factor analysis to identify latent constructs within the new measure. The finalized scale was analyzed for test-retest reliability, internal consistency, and convergent validity.

**Result:**

An exploratory factor analysis yielded a four-factor scale with 23 items. The finalized 23 items had high internal consistency (Cronbach’s alpha = 0.91) and relatively high test-retest reliability (ICC = 0.83). The four subscales had fair internal consistency (Cronbach’s alpha ≥ 0.69) and good test-retest reliability (ICC ≥ 0.61). Convergent validity was weakly supported by the significant positive correlations with the overall question on motivation to work (r ≥ 0.19, p < 0.01). Besides these correlations, only the “Pressure from others” subscale was negatively and significantly correlated with the negative symptoms evaluated using the Positive and Negative Syndrome Scale (r = -0.18, p = 0.04).

**Conclusions:**

This study used factor analysis to develop a new multidimensional scale assessing motivation for competitive employment among persons with severe mental illness. The scale showed acceptable levels of reliability and factor-based and convergent validity. The new measure can be used for measuring the motivation for competitive employment among people with severe mental illness, and it would be useful to identify candidates who are likely to benefit from a certain supported employment program, and to monitor interim progress of the state of participants’ motivation in a program.

## Introduction

Working is a stated goal of people with severe mental illness.[1–3] Most prefer competitive employment, which is defined as a community-based job that pays at least the minimum wage.[4, 5] However, in many countries the competitive employment rate in this group is still low compared to that of the general population.[6–11]

To address this situation, studies have been conducted in several countries, including Japan, to promote effective vocational support, particularly via evidence-based supported employment programs.[12–16] However, around half of the participants in these programs did not gain employment.[14] Although most client factors were shown not to affect evidence-based supported employment,[17] a trial in the United Kingdom suggested that individuals’ low motivation to work was a main cause of low employment outcomes, even in a supported employment program.[18]

Previous studies on factors that facilitate employment in this population have suggested that the motivation to work is related to employment outcomes.[19–21] In keeping with a previous qualitative study, the motivation to work can be defined as the impetus to obtain competitive employment, and is influenced by multiple reasons.[22] In the precedent research on motivation, motivation has been often investigated by categorizing into several factors, such as intrinsic and extrinsic motivation, self-determined motivation and nonself-determined motivation[23, 24]. In the interview for people with mental illness, five categories of thoughts were derived that support motivation for work activities.[22] The participants in the interview wanted to get away from their present state, to gain the feeling of tension and feel a duty to lead their life, to gain confidence and pride, and to regain connections with other people, and they expected to change themselves by working. Both the strength of motivation at baseline and improvement of motivation showed a positive correlation with vocational outcomes in prospective cohort studies,[20, 25] Also, people with severe mental illness who gained competitive employment had higher motivation compared with those who eventually registered in a sheltered workshop.[21] Work motivation also was used as an outcome indicator of the performance of vocational rehabilitation programs.[19]

Given these associations, measuring the motivation for competitive employment among people with severe mental illness is essential to identify candidates who are likely to benefit from certain supported employment programs, and to monitor the interim progress of such programs. However, the instruments used in prior studies to assess the motivation to work were not developed specifically for employment among people with severe mental illness; rather, they were comprised of specific items from existing scales, such as the Quality Life Scale (QLS) [26] or the Worker Role Interview (WRI) [27], and there were studies which used an original questionnaire [22, 25]. The WRI was developed to assess motivation among working persons without mental illness, whose thoughts on working might be different from those of people with severe mental illness. Studies based on part of the QLS or that employed an original questionnaire used only one item to assess whether the person was motivated or not.[20, 21, 28] Also, the WRI and QLS are administered in the context of semi-structured interviews, which makes it difficult to use them in larger samples or in clinical practice. In a prospective cohort study in the US, Reddy et al (2016) also examined an association between employment for people with schizophrenia and their intrinsic and extrinsic motivation using Intrinsic Motivation Inventory (IMI) and Motivators and Barriers to Employment Questionnaire (MBEQ), respectively[25]. However, although the psychometric properties of IMI in schizophrenia sample has been confirmed [23], IMI does not cover extrinsic motivation. In addition, the construct or factor validity of MBEQ appears to be relatively unclear. We could not gain the detailed information on MBEQ since the paper of MBEQ has been currently unpublished according to the Reddy and colleagues’ study (2016). In sum, there is no measure for assessing both subjective work intrinsic and extrinsic motivation for people with severe mental illness.

The purpose of this study was to develop a new measure to comprehensively assess the motivation to attain competitive employment in people with severe mental illness, and to examine the reliability and validity of the new measure. The new tool is expected to be useful to investigate the association between motivation and vocational outcomes, evaluate the effect of vocational rehabilitation programs on participants’ motivation, provide insight into how to increase motivation regarding competitive employment in people with severe mental illness, and prevent these individuals from dropping out of vocational services.

## Materials and methods

### Methods

#### Scale development

First, an item pool was created based on a literature search and a focus group interview of three persons with schizophrenia. The literature review was conducted through PubMed, PsycINFO, and a Japanese academic database (Ichushi), using several relevant keywords (specifically, motivation, employment, work, vocational, mental disorders, mental disabilities, mental illness, and schizophrenia). Language was restricted to English or Japanese. Items mentioned in qualitative studies were included in the item pool,[22, 29–31] as were those from the WRI, a semi-structured interview scale [27] that was used in prior studies to investigate the association between motivation and work.[19] In the focus group interview, two persons with mental illness who were seeking employment, one person with mental illness who had obtained a job through a vocational rehabilitation program. Written informed consent was obtained from the three participants in the focus group. Participants were asked the following questions: What triggered you to pursue work? What improved your willingness to work? What do you want to attain through working? How do you maintain this willingness? Interview notes were used to collect relevant ideas concerning motivation to work. Then, a preliminary scale was constructed based on a systematic item reduction method, the KJ method.[32] The scale and items were refined based on feedback from a researcher in the field of vocational rehabilitation and three peer workers with schizophrenia.

#### Validation study

To refine the item composition and test the reliability and validity of the final draft of the 38-item scale developed in the procedure described above, the instrument was administered to a cross-section of people with severe mental illness from January 2015 to January 2016. The inclusion criteria for subjects in this cross-sectional survey were: 1) diagnosis of schizophrenia/schizoaffective disorder; 2) age 20 years (the age of majority in Japan) or older; 3) and registration in any of four job transition support centers or six psychiatric day-care centers with vocational rehabilitation programs located in Miyagi, Kanagawa, Fukuoka, Tokyo, Chiba, Kyoto, Hiroshima, and Kumamoto prefectures. With regards to vocational rehabilitation for persons with disabilities in Japan, three main employment services, including job transition support, continuous employment Type-A, continuous employment Type-B, are defined under the Comprehensive Services and Supports for Persons with Disabilities Act[33]. Continuous employment Type-A and Type-B are both types of sheltered workshops. Continuous employment Type-B does not provide employment contracts and usually pays below the minimum wage, while continuous employment Type-A provides an employment contract for users and pays them above minimum wage with a government subsidy. Job transition support aims to obtain competitive employment for users by providing a variety of employment services. In addition to job transition support centers, some psychiatric day-care centers, outpatient services, and visiting nursing centers also provide employment support.

A written description of the study was given to all participants and informed consent was obtained. A self-report questionnaire was administered to all participants. Symptom assessments were completed by a psychiatrist or clinical psychologist at each day-care center. At vocational rehabilitation centers with no psychiatrist or clinical psychologist, a clinical psychologist of the National Institute of Mental Health completed the assessments. Two weeks after the first cross-sectional survey, the same provisional measure was administered to participants who agreed to complete the questionnaire again, in order to confirm test-retest reliability.

All data were collected after receiving approval from the research ethics committee of the Graduate School of Medicine, The University of Tokyo, and the ethical committee of the National Center of Neurology and Psychiatry.

### Materials

The self-report questionnaire included a provisional scale on work motivation developed in this study, a question assessing overall work motivation (How strongly do you wish to begin working now?), the SF-8^™^, and questions on demographic information. Items on the provisional scale on work motivation and the question assessing overall work motivation were rated on 4-point Likert scales ranging from 1 (strongly disagree) to 4 (strongly agree). We calculated subscale scores of the scale on work motivation by calculating the mean of the items within each subscale. At the two-week follow-up, only the provisional motivation measure was conducted again in order to investigate test-retest reliability.

1. Demographic variables
  Demographic variables, including age, sex, work experience within the past year (i.g., Have you obtained employment more than once within the past year, including part-time job?), education, living status, and disability pensions, were obtained in the self-report questionnaire.
2. SF-8
  The SF-8 is an 8-item self-report measure that subjectively assesses health-related quality of life.[34] Each question evaluates an independent aspect of quality of life: physical functioning, role physical, bodily pain, general health, vitality, social functioning, role emotional, and mental health. Using the scoring rules, the summary scores for physical and mental health can be obtained from 8 items. The reliability and validity of the Japanese version have been confirmed.[35] We obtained permission to use SF-8 for this study.
3. Positive and Negative Syndrome Scale (PANSS)
  The Positive and Negative Syndrome Scale (PANSS) was used for symptom assessment.[36] The PANSS is a 30-item clinician-rated measure that assesses clinical psychiatric symptoms subdivided into three categories: positive scale, negative scale, and general psychopathology scale. Items are rated on 7-point Likert scales ranging from 1 (absent) to 7 (extremely severe). Symptoms of each participant are rated within 1 month after his or her questionnaire is completed. The inter-rater reliability and the internal consistency of the Japanese version have been confirmed.[37]

### Statistical analysis

#### Exploratory factor analysis

Prior to the factor analysis, seven items (#3, #6, #13, #18, #28, #29, #36) that were highly correlated with other items were removed (r > 0.7, p < 0.05). We remained the item which was the name of the team with more labels in KJ method. Remaining items were subjected to an exploratory factor analysis to identify latent constructs within the new measure. The analysis was performed using principal factor analysis, with promax rotation, as it was considered that the factors would be correlated. The number of factors to be extracted was determined by examining the eigenvalue and the scree plot. The eigenvalue cutoff was set at 1.0. After dropping items with factor loadings less than 0.4, iterative factor analysis was performed.

#### Convergent and divergent validity

Convergent and divergent validity for subscale scores and total score of the new motivation scale were analyzed by calculating the Spearman’s product-moment correlation to test the associations with other measures. Specifically, we compared the scores of the new motivation scale with those of the PANSS, SF-8, and the question assessing overall work motivation.

#### Reliability

Cronbach’s alpha for each subcategory and the total score were calculated to test internal consistency, with a criterion of 0.70 taken as indicating good internal consistency.[38] Test-retest reliability was evaluated using intra-class correlation (ICC) for the total score and the subscale total scores. All analyses were performed using Stata version 13.

## Results

### Scale development

Based on the literature review and focus group interview, a 108-item pool was generated. A tentative draft consisting of 39 items was created from the item pool based on the KJ method [32], a systematic item reduction approach. The KJ method involves four steps 1) label making, 2) label grouping, 3) chart making, 4) written or verbal explanation. In the first step, the label making step, information based upon observations relevant to the problem is written on note cards or specially designed self-adhesive labels, so that each note card contains only one concept. In this study, 108-items based on the literature review and focus group interview were written down on note cards. Then, in the next step, label grouping, the note cards were grouped into "teams" and named each team. In the third step, the order of the placement of these teams was arranged according to the contents. The teams which seem to be closer in meaning to each other were placed closer and vice versa. In the fourth step, written or verbal explanations of findings through these precedent steps. In this study, we aimed to create the tentative questionnaire utilizing this item reduction approach, and we did not publish the result as other written explanations before this research report. These 39 items were tentatively divided into 12 teams related to motivation: new encounters, utilization of one’s own experiences, financial independence, value of working, satisfying private time, social support, recognition by others, new lifestyle, appreciation by others, sense of accomplishment, acquisition of new abilities, and new role.

Based on feedback from a researcher in the field of vocational rehabilitation and three peer workers with schizophrenia, one item (“I want to pursue my career goals”) was removed from the draft due to its ambiguity. The final draft with 38 items was created and used in the following validation study.

### Validation study

A total of 145 individuals participated, 117 of whom also completed the questionnaire for motivation to work at the second administration to provide test-retest reliability data. The data of 136 individuals without any missing data in the questionnaire for motivation to work and PANSS were included for analysis. Six participants did not respond to some items in the questionnaire for motivation to work, and three participants did not receive PANSS assessment. For test-retest reliability data, 104 individuals without any missing data in the baseline of motivation to work and the retest of motivation to work were included. The characteristics of the participants are shown in Table 1. There was no significant difference in any of these characteristics between those who responded to the retest survey and those who did not (p > 0.05).

**Table 1:** Baseline demographic characteristics (N = 136).

	n (%)	Average (SD)
**Age (years)**		38.6 (9.1)
**Sex**		
Men	89 (65.4)	
Women	47 (34.6)	
**Education (years)**		
Less than 12	13 (9.6)	
12–16	84 (61.8)	
16 or more	39 (28.7)	
**Employment experience within the past year**		
None	84 (61.8)	
More than one	52 (38.2)	
**Living status**		
Living alone	28 (20.6)	
With family members	96 (70.6)	
Other	12 (8.8)	
**Discontinuation of treatment for more than 3 months within the past year**		
Yes	2 (1.5)	
No	134 (98.5)	
**Hospitalization for more than a month within the past year**		
Yes	21 (15.4)	
No	115 (84.6)	
**Disability pension**		
Yes	89 (65.4)	
No	47 (34.6)	

#### Exploratory factor analysis

Prior to factor analysis, seven items (#3, #6, #13, #18, #28, #29, and #36) were deleted as they were highly correlated with other items (r > 0.7, p < 0.05). The exploratory factor analysis yielded a four-factor model. Repeating the analysis with the four-factor model, 23 items with loadings greater than 0.4 were retained. The pattern matrix is shown in Table 2. The extracted Factor 1 consisted of 10 items that could be interpreted as “Relationships With Others.” The items highly loaded on this factor were categorized separately into “satisfying private time,” “recognition by others,” and “appreciation by others” in the systematic item reduction process. The extracted Factor 2 consisted of seven items that could be interpreted as “Personal Growth.” The items categorized into “utilization of one’s own experiences,” “value of working,” “sense of accomplishment,” “acquisition of new abilities,” and “new role” highly loaded on this factor. The extracted Factor 3 consisted of four items that could be interpreted as “Change Of Lifestyle.” The items categorized into “new encounters” were highly loaded on this factor. The extracted Factor 4 consisted of two items that could be interpreted as “Pressure From Others.”

**Table 2:** Rotated factor loadings and percentage variance explained (N = 136).

#Item	Item wording	1	2	3	4
	Factor 1—Relationships with others				
#15	I want more opportunities to be appreciated by others	**0.88**	0.00	-0.01	-0.17
#14	I want more opportunities to be appraised by others	**0.84**	-0.06	0.12	-0.11
#19	I want to be trusted more by others	**0.78**	-0.02	0.13	0.00
#23	I want to be approved of by my family	**0.74**	-0.13	0.03	0.08
#21	I want opportunities to have my opinions be heard in the workplace	**0.69**	0.29	-0.34	-0.02
#5	I want new friends	**0.53**	0.10	0.10	0.06
#20	I want to expand my range of activities	**0.50**	0.08	0.24	-0.04
#22	I want opportunities to be relied on by colleagues	**0.48**	0.39	-0.01	-0.08
#4	I want to spend more time with others	**0.47**	0.01	0.21	-0.03
#24	I want colleagues to provide some support in my personal life	**0.40**	0.23	-0.16	0.23
	Factor 2 –Personal growth				
#27	I want to acquire new skills	-0.02	**0.73**	-0.10	0.10
#16	I want to utilize my skills	0.00	**0.72**	-0.03	0.14
#1	I think that working is interesting	-0.12	**0.58**	0.00	-0.13
#26	I want to be appointed to a responsible position	0.16	**0.52**	0.09	-0.02
#17	I want to utilize my experiences	0.28	**0.46**	0.03	0.02
#34	I want to experience some achievement in the workplace	0.19	**0.46**	0.17	0.04
#2	I want to gain the feeling of tension to some extent	-0.13	**0.43**	0.41	-0.11
	Factor 3 –Change of lifestyle				
#11	I want something to do everyday	-0.02	0.00	**0.77**	0.07
#12	I want to lead a well-regulated life	0.07	-0.13	**0.73**	0.04
#9	I want to lead a lively life	0.21	0.01	**0.55**	0.03
#10	I want to acquire a new role	0.01	0.37	**0.41**	0.09
	Factor 4 –Pressure from others				
#33	I am told by people other than my family members that I should work	-0.25	0.16	0.11	**0.90**
#30	I am told by my family members that I should work	0.26	-0.11	-0.02	**0.55**
	Variance explained (%)	58.0	46.0	35.5	19.5

Note. |Factor loadings| > 0.40 are in boldface.

# Item number in the tentative questionnaire used in this study

#### Convergent and divergent validity

The correlations between the new measure and other measures are shown in Table 3. The total score and each subscale score for the new motivation measure showed weak correlations with the question assessing overall work motivation (r ≤ 0.31, p < 0.01). The “Pressure From Others” subscale was negatively and significantly correlated with the PANSS negative symptom subscale, although the correlation coefficient value was small (r = -0.18, p = 0.04). We did not find other significant correlations between measures.

**Table 3:** Pearson’s correlations of the new scale scores to SF-8, PANSS and the score on overall motivation to work (N = 136).

	Mean^a)^	SD^a)^	SF-8^b)^		PANSS			Overall motivation
			Physical health	Mental health	Positive symptoms	Negative symptoms	General psychopathology	
Total score	68.6	11.2	-0.01	0.07	0.00	-0.05	0.09	0.30**
Relationships with others	29.6	6.0	-0.05	0.12	0.06	0.02	0.16	0.19**
Personal growth	20.4	3.9	0.07	-0.05	-0.01	-0.08	0.03	0.25**
Change of lifestyle	13.4	2.1	-0.05	0.12	0.00	-0.03	0.06	0.31**
Pressure from others	5.3	1.8	0.03	0.05	-0.15	-0.18*	-0.12	0.30**

^a)^ Means and standard deviations were calculated using raw scores.

^b)^ Correlations with SF-8 were calculated using data of 135 participants without missing data in all items of SF-8

* Correlation is significant at the 0.05 level.

** Correlation is significant at the 0.01 level.

#### Reliability

The Cronbach’s alpha and ICCs for the entire scale score and the subscale total scores are shown in Table 4. The alpha value for the total score and each subscale score were above 0.70, indicating acceptable internal consistency, except the “Pressure From Others” subscale. The ICC for the entire scale score was 0.83 and those of its subscales were between 0.61 and 0.85, indicating moderate to high agreement.

**Table 4:** Cronbach’s alpha (N = 136), intraclass correlations between test and retest (n = 104).

	Cronbach’s alpha	ICC	Mean (SD)
Total score	0.91	0.83	68.6 (11.2)
Relationship with others	0.90	0.85	2.96 (0.60)
Personal Growth	0.82	0.80	2.91 (0.56)
Change of lifestyle	0.77	0.61	3.36 (0.53)
Pressure from others	0.69	0.79	2.63 (0.91)

## Discussion

Through use of a literature search, focus-group interview, and tests for psychometric properties, this study developed a new measure to assess the motivation for competitive employment among people with severe mental illness. The new measure consisted of 23 items and was found to have good internal consistency and reasonable test-retest reliability. The alpha value for total score and each subscale were above 0.70, indicating acceptable internal consistency, except for the “Pressure From Others” subscale. The alpha value for this subscale was 0.69, which was close to the acceptable level, but still indicated uncertain consistency. Future studies should reinvestigate the reliability of each subscale. The ICC for the entire scale score and its subscales indicated moderate to high test-retest reliability.

Four factors were extracted from the exploratory factor analysis of the 23 items. The factors seem to be consistent with constructs that emerged from prior qualitative studies,[22, 29, 31] which may support the content validity of the new measure. In the interview conducted in the welfare facility in Japan, five categories of thoughts were derived that support motivation for work activities. The participants in the interview wanted to get away from their present state, to gain the feeling of tension and feel a duty to lead their life, to gain confidence and pride, and to regain connections with other people, and they expected to change themselves by working.[22] Three extracted factors, “Relationships With Others,” “Personal Growth,” and “Change Of Lifestyle,” seemed consistent with these thoughts. The two factors “Personal Growth” and “Change Of Lifestyle” were also in accordance with the results of the other interview studies conducted by the San Francisco Community Mental Health Services to examine the phenomenon of the lived experience of work, focusing on the time after the individual decided to begin working.[29] The authors listed five motivators: “Work Was a Contributor to the Person’s Identity,” “External Motivators Drew the Person to Work,” “Work Was an Antidote to the Person’s Problems,” “Work Was a Conduit to Personal Growth and the Development of Competencies,” and “Work Provided Various Types of Personal Gain.” In another interview study in the United States that explored respondents’ perspectives on employment and its relationship to their vocational recovery, the interviewees mentioned that financial reasons fueled their motivation to find a job or continue working, and participants’ motivation for vocational success was also apparent when they described career goals they hoped to accomplish in the future.[31] The extracted factor “Personal Growth” might accord with such desires to accomplish career goals. It should be noted that no items which directly mentioned financial incentives remained after the exploratory factor analysis, although two related items (#7, #8) existed in the preliminary questionnaire. For these two items, 72.8% respondents selected the maximum score on #7, and 58.8% of respondents selected the maximum score on #8. Compared to other items, the means of these two scores were higher, and the variances were small. It might be possible that their factor loadings became small due to their small variances. Although there is no item directly mentioning financial incentives in the new measure, considering the fact of the high endorsement proportions of these two items, it might be desirable to obtain information concerning the participants’ financial conditions separately when the new measure will be used in a study. Also, national disability benefits statutes might affect the employment situation of people with disability. In the meta-analysis to assess the impact of site-level moderators on the effectiveness of an evidence-based rehabilitation program, the intervention arms experienced a 3% decrease in the probability of competitive employment for every one point increment in OECD-developed indices which assess the generosity, ease of access to, permanence of benefits, accessibility of vocational rehabilitation and incentives provided for joining the workforce. [39] It might also be that the high rate of cohabitation with parents in Japan influence the relationship between the motivation to work and the financial incentives. The cohabitation rate was 70.6% in this study, which was similarly high in the national survey of persons with mental disorders (67.8%)[40], it was higher compared to the cohabitation rate with caregivers reported in the US (45%) [41]. According to the result of the study on caregivers and siblings in 866 households belonging to 27 affiliate family groups under a prefectural-level family group association in Japan, the household incomes were higher than 20 thousand dollars in 86.3% of respondents[42]. It might be possible that there are people who want to become financially independent, but due to their living environment in Japan, such feelings might not be directly related to the motivation to work.

Significantly positive correlations were observed between the question assessing overall motivation to work and the total and subscale scores of the new measure, partly suggesting the convergent validity of the new measure. However, we used the one original question to test the convergent validity rather than an alternative scale related to work motivation. Therefore, further studies may need to confirm the association between this measure and other motivation scales. In terms of another correlation, the “Pressure From Others” subscale, which can be considered to represent extrinsic motivation, was negatively and significantly correlated with the PANSS negative symptom subscale. Although the correlation coefficient value between “Pressure From Others” subscale and PANSS negative symptom subscale was small, this result of this negative correlation might suggest that as negative symptoms are reduced, people will have the energy to act and will care more about what others expect from them. Other three subscales in the new measure, which are considered to represent intrinsic motivation, did not show any significant correlations with the PANSS subscale. This result is not consistent with a previous study that suggested that the negative syndrome had a significant negative correlation with intrinsic motivation. In a cross-sectional survey in the US, Saperstein et al (2011) examined an association between employment for people with schizophrenia or schizoaffective disorder and their intrinsic motivation using QLS. In the study, negative symptoms assessed using SANS and intrinsic motivation correlated significantly (r = -0.59, p < 0.01).[28] It is probable that the negative correlation of negative symptoms to extrinsic motivation is stronger than to intrinsic motivation, and correlations between intrinsic motivation and negative symptoms just could not be detected due to the small number of participants. With regard to other correlations, the total score and all subscale scores did not correlate with the subjective assessment of quality of life, as measured using the SF-8. This result did not match those of previous studies. Several qualitative research studies suggested that persons who are dissatisfied with their present state tend to voice their desire to work,[22, 30] so in our study the scores of the SF-8 and the new measure should have been negatively correlated. However, the present null findings may be reasonable if the motivation to work is independent of symptoms and quality of life in specific situations, such as in job transition support centers. The participants in this study are registered in job transition support centers or in vocational rehabilitation programs in day-care centers, so their motivation might be affected by the environment.

### Study limitations and strengths

Some limitations should be considered. First, the sample in this study was limited to persons with schizophrenia or schizoaffective disorders. The psychometric properties should be further examined in other disorders, including severe depression and bipolar disorder. For example, there was the finding in Japan that there was a difference in employment situation between workers with schizophrenia and workers with other mental illnesses. According to a cross-sectional study conducted in 2015, 74.3% of workers with schizophrenia disclosed their illness to their employers, while 57.9% of workers with bipolar disorders, and 78.5% of workers with other mental illness disclosed their illness to their employers. [43] Also, previous research showed that employment outcomes were significantly related to depression severity among people with affective disorders.[44] Therefore, it is probable that the psychometric properties of this work motivation scale and employment outcomes would be affected by depression severity. Second, the study participants were users of job transition support centers and psychiatric day-care centers, and this study did not include people in sheltered workshops, who may also want to gain competitive employment.[2] The motivation to work in people who use rehabilitation service centers or psychiatric day-care centers may be higher than in those at sheltered workshops. According to the questionnaire survey conducted in 2008, 75.0% of users in job transition support centers thought that working would be beneficial for themselves, while 58.8% of users in sheltered-workshops thought in that way.[2] In other words, our measure was developed based on people with mental illness who have a relatively high motivation to gain competitive work. Third, the numbers of participants both in the focus group interview and the validation study were small. For a focus group interview to generate an item pool, usually 6 to 8 people participate, and for an 18-item scale, more than 380 participants are needed. Therefore, the validity of the new scale should be further investigated with a larger sample. Confirmatory factor analysis should be performed to ascertain the structure of the new measure. Fourth, we should investigate the factor structure with another sample by confirmatory factor analysis. Lastly, further research is required to determine how the scores in the new measure relate to employment. Longitudinal studies need to be carried out to ascertain the relationship between the score of this new measure and the employment outcomes. The newly developed, self-administered measure can be more easily completed than a semi-structure interview measure like the WRI. Also, since the measure is relatively brief and should not burden participants, it should be feasible to use it in research or practice in the future. One important potential application of the new measure is in longitudinal research assessing the effect of vocational rehabilitation methods on participants’ motivation. Since a primary strength of the new measure is its multidimensionality, in contrast to a single question asking whether a person is motivated or not, it may provide a detailed description of which domain of motivation is associated with a respondent’s attitude toward competitive employment. This should assist support staff in helping users maintain their motivation, preventing their drop-out from vocational rehabilitation programs.

## Conclusions

The newly developed measure presented here can be used to assess the motivation for competitive employment among people with severe mental illness, and this study provides preliminary evidence for its reliability and validity. It can be a useful tool to better understand the role of motivation, and it would be useful to identify candidates who are likely to benefit from a certain supported employment program, and to monitor the interim changes of motivation in a program.

## Supporting information

S1 Table List of terms appeared in the focus group interview. (DOCX)

S2 Table A tentative scale used in this study (English translation). This is the scale which was used in the study. After collecting data using this scale, the exploratory factor analysis was performed.
(DOCX)

S3 Table A tentative scale used in this study (Japanese original version). This is the scale which was used in the study. After collecting data using this scale, the exploratory factor analysis was performed.
(DOCX)

S4 Table Correlation matrix used in the exploratory factor analysis and p-value. Prior to factor analysis, seven items (#3, #6, #13, #18, #28, #29, and #36) were deleted as they were highly correlated with other items.
(DOCX)

S5 Table Frequency of response to each item of the new scale. Frequency of response to each item, including the items which were removed before factor analysis.
(DOCX)

S1 Fig Process of creating the new measure. (DOCX)
