# Supplementary material for: Development of a scale to assess motivation for competitive employment among persons with severe mental illness
Source: PLoS One. 2018 Oct 2;13(10):e0204809. doi: 10.1371/journal.pone.0204809 (PMC6168136; doi:10.1371/journal.pone.0204809)
Supplement: S1 Table — (DOCX) [file pone.0204809.s001.docx]

S1 Table. List of terms appeared in the focus group interview

| "decide what to do in my work by myself" |
| --- |
| "support from people whom I'm familiar with (besides my own family)" |
| "do the job in which I can feel high satisfaction" |
| "gain the sense of accomplishment" |
| "feel satisfaction by trying hard" |
| "gain some experiences that I've done well" |
| "acquire new skills" |
| "acquire skills to solve problems" |
| "acquire an ability to make a decision" |
| "improve an ability to make an effort" |
| "acquire an ability to do something with other people" |
| "live following a regular routine" |
| "vary the pace of my life" |
| "gain social status" |
| "change their (other close people besides my family) perception of me" |
| "make it easier to meet people whom I lost touch after becoming ill" |
| "their (other close people besides my family) attitudes towards me" |
| "realize their (other close people besides my family) wishes that I obtain employment" |
| "make new friends" |
| "make new acquaintances" |
| "have more time to work with other people" |
| "have time to work with other people" |
| "utilze my skills" |
| "gain the trust of my family" |
| "get recognition from my family" |
| "make my family happy" |
| "gain the trust of close people besides my family" |
| "get recognition from close people besides my family" |
| "get money to use for my hobbies" |
| "lead an independent life" |
| "will not receive income security" |
| "do the work that I can enjoy" |
| "work in the field in which I'm interested" |
| "my opinions are respected" |
| "listen to what I say" |
| "feel safe while working" |
| "colleagues expect contributions of me" |
| "have a boss with whom I can ask for advice" |
| "do the job in which I contribute to the society" |
| "do the job in which I contribute to other people" |
| "have been able to come to a vocational rehabilitation service center continuously" |
| "told by a medical staff, 'You can work' " |
| "become able to work for five days a week" |
| "have something to do for two to three days a week" |
| "spend a lot of time in a day for working" |
