# Supplementary material for: Development of a scale to assess motivation for competitive employment among persons with severe mental illness
Source: PLoS One. 2018 Oct 2;13(10):e0204809. doi: 10.1371/journal.pone.0204809 (PMC6168136; doi:10.1371/journal.pone.0204809)
Supplement: S2 Table — This is the scale which was used in the study. After collecting data using this scale, the exploratory factor analysis was performed. (DOCX) [file pone.0204809.s002.docx]

S2 Table. A tentative scale used in this study (English translation)

| Would you like to tell us the reasons why you want to work? Do the items below apply? Please circle the answer which apply you most. | | | | | |
| --- | --- | --- | --- | --- | --- |
| # |  | Strongly disagree | Disagree a little | Agree a little | Strongly agree |
| 1 | I think that working is interesting | 1 | 2 | 3 | 4 |
| 2 | I want to feel tension to some extent | 1 | 2 | 3 | 4 |
| 3 | I want more opportunities to talk with other persons | 1 | 2 | 3 | 4 |
| 4 | I want to spend more time with other persons | 1 | 2 | 3 | 4 |
| 5 | I want new friends | 1 | 2 | 3 | 4 |
| 6 | I want new acquaintances | 1 | 2 | 3 | 4 |
| 7 | I want to lead a financially independent life | 1 | 2 | 3 | 4 |
| 8 | I want to have more money to spend on what I like | 1 | 2 | 3 | 4 |
| 9 | I want to lead a lively life | 1 | 2 | 3 | 4 |
| 10 | I want to acquire a new role | 1 | 2 | 3 | 4 |
| 11 | I want something to do everyday | 1 | 2 | 3 | 4 |
| 12 | I want to lead a well-regulated life | 1 | 2 | 3 | 4 |
| 13 | I want to be approved by other persons (not family members) | 1 | 2 | 3 | 4 |
| 14 | I want more opportunities to be appraised by other persons | 1 | 2 | 3 | 4 |
| 15 | I want more opportunities to be appreciated by others | 1 | 2 | 3 | 4 |
| 16 | I want to utilize my skill | 1 | 2 | 3 | 4 |
| 17 | I want to utilize my experiences | 1 | 2 | 3 | 4 |
| 18 | I want that my good points would be recognized by others | 1 | 2 | 3 | 4 |
| 19 | I want to be trusted more by other persons | 1 | 2 | 3 | 4 |
| 20 | I want to expand my range of activity | 1 | 2 | 3 | 4 |
| 21 | I want opportunities that my opinions are heard in the workplace | 1 | 2 | 3 | 4 |
| 22 | I want opportunities to be relied on by colleagues | 1 | 2 | 3 | 4 |
| 23 | I want to be approved by my family | 1 | 2 | 3 | 4 |
| 24 | I want some support for my personal lives by colleagues | 1 | 2 | 3 | 4 |
| 25 | I want some opportunities to make an effort for | 1 | 2 | 3 | 4 |
| 26 | I want to be appointed to a responsible position | 1 | 2 | 3 | 4 |
| 27 | I want to acquire new skills | 1 | 2 | 3 | 4 |
| 28 | I want to learn new things | 1 | 2 | 3 | 4 |
| 29 | I want that the confidence from my family grows | 1 | 2 | 3 | 4 |
| 30 | I am told by my family that I should work | 1 | 2 | 3 | 4 |
| 31 | Working is important for myself | 1 | 2 | 3 | 4 |
| 32 | I feel pressure that I should work | 1 | 2 | 3 | 4 |
| 33 | I am told by other people except my family that I should work | 1 | 2 | 3 | 4 |
| 34 | I want to experience some achievement in the workplace | 1 | 2 | 3 | 4 |
| 35 | I want to be more confident | 1 | 2 | 3 | 4 |
| 36 | I want to feel the sense of accomplishment | 1 | 2 | 3 | 4 |
| 37 | I want my social position | 1 | 2 | 3 | 4 |
| 38 | I want to contribute for the society | 1 | 2 | 3 | 4 |
| # Item number in the tentative questionnaire used for this study | | | | | |
