# Supplementary material for: Development of a scale to assess motivation for competitive employment among persons with severe mental illness
Source: PLoS One. 2018 Oct 2;13(10):e0204809. doi: 10.1371/journal.pone.0204809 (PMC6168136; doi:10.1371/journal.pone.0204809)
Supplement: S3 Table — This is the scale which was used in the study. After collecting data using this scale, the exploratory factor analysis was performed. (DOCX) [file pone.0204809.s003.docx]

|  | S3 Table. The tentative scale used in this study (Japanese) | | | | | | |  |
| --- | --- | --- | --- | --- | --- | --- | --- | --- |
|  | あなたが就職を希望する動機についてお尋ねします。 以下の項目はあなたの就職したい理由に当てはまりますか。もっとも近い回答に◯をつけてください。 | | | | | | | |
|  |  |  |  | |  | | |  |
| # |  | まったく当てはまらない | | やや当てはまらない | | やや当てはまる | 非常に当てはまる | |
| 1 | 仕事をすることは楽しいと思う | 1 | | 2 | | 3 | 4 | |
| 2 | 仕事をすることで，ほどよい緊張感を得たい | 1 | | 2 | | 3 | 4 | |
| 3 | 仕事をすることで，他の人と話す時間を増やしたい | 1 | | 2 | | 3 | 4 | |
| 4 | 仕事をすることで，他の人と一緒にいる時間を増やしたい | 1 | | 2 | | 3 | 4 | |
| 5 | 仕事を通じて，新しい友人が欲しい | 1 | | 2 | | 3 | 4 | |
| 6 | 仕事を通じて，知り合いが欲しい | 1 | | 2 | | 3 | 4 | |
| 7 | 経済的に自立した生活を送りたい | 1 | | 2 | | 3 | 4 | |
| 8 | 趣味など，好きなことに使うお金を得たい | 1 | | 2 | | 3 | 4 | |
| 9 | メリハリのある生活を送りたい | 1 | | 2 | | 3 | 4 | |
| 10 | 自分の新たな役割を得たい | 1 | | 2 | | 3 | 4 | |
| 11 | 毎日やるべきことを得たい | 1 | | 2 | | 3 | 4 | |
| 12 | 規則正しい生活を送りたい | 1 | | 2 | | 3 | 4 | |
| 13 | 仕事をすることで，家族以外の周りの人に認められたい | 1 | | 2 | | 3 | 4 | |
| 14 | 人から感謝される機会を増やしたい | 1 | | 2 | | 3 | 4 | |
| 15 | 人から褒められる機会を増やしたい | 1 | | 2 | | 3 | 4 | |
| 16 | 自分のスキル(技能)を仕事に活かしたい | 1 | | 2 | | 3 | 4 | |
| 17 | 自分の人生経験を仕事に活かしたい | 1 | | 2 | | 3 | 4 | |
| 18 | 自分の良い点を認められたい | 1 | | 2 | | 3 | 4 | |
| 19 | 仕事をすることで，家族以外の周りの人の信頼を増したい | 1 | | 2 | | 3 | 4 | |
| 20 | 職場に通うことで行動範囲を広げたい | 1 | | 2 | | 3 | 4 | |
| 21 | 仕事上で自分の意見を聞いてもらう機会を得たい | 1 | | 2 | | 3 | 4 | |
| 22 | 職場の人から頼られる機会を得たい | 1 | | 2 | | 3 | 4 | |
| 23 | 仕事をすることで，家族の人に認められたい | 1 | | 2 | | 3 | 4 | |
| 24 | 職場の人から生活面でのサポートを得たい | 1 | | 2 | | 3 | 4 | |
| 25 | 努力する機会を得たい | 1 | | 2 | | 3 | 4 | |
| 26 | 責任のある立場に就きたい | 1 | | 2 | | 3 | 4 | |
| 27 | 仕事を通じて新たなスキル(技能)を得たい | 1 | | 2 | | 3 | 4 | |
| 28 | 仕事を通じて新たなことを学びたい | 1 | | 2 | | 3 | 4 | |
| 29 | 仕事をすることで，家族からの信頼を増したい | 1 | | 2 | | 3 | 4 | |
| 30 | 就職した方がよいと，家族から言われる | 1 | | 2 | | 3 | 4 | |
| 31 | 仕事をするのは自分にとって重要な事だから | 1 | | 2 | | 3 | 4 | |
| 32 | 仕事をしなければいけないという気持ちを感じる | 1 | | 2 | | 3 | 4 | |
| 33 | 就職をした方がよいと，家族以外の周りの人に言われる | 1 | | 2 | | 3 | 4 | |
| 34 | 仕事上での目標達成を経験したい | 1 | | 2 | | 3 | 4 | |
| 35 | 仕事をすることで自信をつけたい | 1 | | 2 | | 3 | 4 | |
| 36 | 仕事を通じて達成感を得たい | 1 | | 2 | | 3 | 4 | |
| 37 | 社会的地位を得たい | 1 | | 2 | | 3 | 4 | |
| 38 | 仕事を通じて社会の役に立ちたい | 1 | | 2 | | 3 | 4 | |
|  | # Item number in the tentative questionnaire used for this study | |  | |  | | |  |
