# Supplementary material for: Development of a scale to assess motivation for competitive employment among persons with severe mental illness
Source: PLoS One. 2018 Oct 2;13(10):e0204809. doi: 10.1371/journal.pone.0204809 (PMC6168136; doi:10.1371/journal.pone.0204809)
Supplement: S4 Table — Prior to factor analysis, seven items (#3, #6, #13, #18, #28, #29, and #36) were deleted as they were highly correlated with other items. (DOCX) [file pone.0204809.s004.docx]

S4 Table. Correlation matrix used in the exploratory factor analysis and p-value

|  | #1 | | #2 | #3 | #4 | #5 | #6 | #7 | #8 | #9 | #10 | #11 | #12 | #13 | #14 | #15 | #16 | #17 | #18 | #19 | #20 | #21 | #22 | #23 | #24 | #25 | #26 | #27 | #28 | #29 | #30 | #31 | #32 | #33 | #34 | #35 | #36 | #37 | #38 |
| --- | --- | --- | --- | --- | --- | --- | --- | --- | --- | --- | --- | --- | --- | --- | --- | --- | --- | --- | --- | --- | --- | --- | --- | --- | --- | --- | --- | --- | --- | --- | --- | --- | --- | --- | --- | --- | --- | --- | --- |
| #1 | 1.00 | |  |  |  |  |  |  |  |  |  |  |  |  |  |  |  |  |  |  |  |  |  |  |  |  |  |  |  |  |  |  |  |  |  |  |  |  |  |
| #2 | 0.38 | | 1.00 |  |  |  |  |  |  |  |  |  |  |  |  |  |  |  |  |  |  |  |  |  |  |  |  |  |  |  |  |  |  |  |  |  |  |  |  |
| **#3^a^** | 0.21 | | 0.30 | 1.00 |  |  |  |  |  |  |  |  |  |  |  |  |  |  |  |  |  |  |  |  |  |  |  |  |  |  |  |  |  |  |  |  |  |  |  |
| #4 | 0.21 | | 0.29 | **0.78*** | 1.00 |  |  |  |  |  |  |  |  |  |  |  |  |  |  |  |  |  |  |  |  |  |  |  |  |  |  |  |  |  |  |  |  |  |  |
| #5 | 0.14 | | 0.24 | 0.64 | 0.68 | 1.00 |  |  |  |  |  |  |  |  |  |  |  |  |  |  |  |  |  |  |  |  |  |  |  |  |  |  |  |  |  |  |  |  |  |
| **#6^a^** | 0.09 | | 0.27 | 0.65 | 0.61 | **0.82*** | 1.00 |  |  |  |  |  |  |  |  |  |  |  |  |  |  |  |  |  |  |  |  |  |  |  |  |  |  |  |  |  |  |  |  |
| #7 | 0.12 | | 0.13 | 0.13 | 0.06 | 0.26 | 0.30 | 1.00 |  |  |  |  |  |  |  |  |  |  |  |  |  |  |  |  |  |  |  |  |  |  |  |  |  |  |  |  |  |  |  |
| #8 | 0.04 | | 0.15 | 0.13 | 0.09 | 0.22 | 0.25 | 0.45 | 1.00 |  |  |  |  |  |  |  |  |  |  |  |  |  |  |  |  |  |  |  |  |  |  |  |  |  |  |  |  |  |  |
| #9 | 0.02 | | 0.42 | 0.36 | 0.30 | 0.31 | 0.35 | 0.32 | 0.32 | 1.00 |  |  |  |  |  |  |  |  |  |  |  |  |  |  |  |  |  |  |  |  |  |  |  |  |  |  |  |  |  |
| #10 | 0.24 | | 0.40 | 0.33 | 0.35 | 0.32 | 0.31 | 0.22 | 0.18 | 0.39 | 1.00 |  |  |  |  |  |  |  |  |  |  |  |  |  |  |  |  |  |  |  |  |  |  |  |  |  |  |  |  |
| #11 | 0.13 | | 0.33 | 0.40 | 0.35 | 0.32 | 0.36 | 0.29 | 0.11 | 0.46 | 0.53 | 1.00 |  |  |  |  |  |  |  |  |  |  |  |  |  |  |  |  |  |  |  |  |  |  |  |  |  |  |  |
| #12 | 0.03 | | 0.34 | 0.33 | 0.25 | 0.30 | 0.33 | 0.35 | 0.18 | 0.55 | 0.27 | 0.58 | 1.00 |  |  |  |  |  |  |  |  |  |  |  |  |  |  |  |  |  |  |  |  |  |  |  |  |  |  |
| **#13^a^** | 0.07 | | 0.22 | 0.34 | 0.36 | 0.42 | 0.40 | 0.24 | 0.32 | 0.36 | 0.35 | 0.36 | 0.35 | 1.00 |  |  |  |  |  |  |  |  |  |  |  |  |  |  |  |  |  |  |  |  |  |  |  |  |  |
| #14 | 0.06 | | 0.24 | 0.40 | 0.40 | 0.46 | 0.51 | 0.28 | 0.27 | 0.42 | 0.40 | 0.31 | 0.32 | **0.74*** | 1.00 |  |  |  |  |  |  |  |  |  |  |  |  |  |  |  |  |  |  |  |  |  |  |  |  |
| #15 | 0.11 | | 0.18 | 0.44 | 0.41 | 0.44 | 0.44 | 0.21 | 0.32 | 0.34 | 0.40 | 0.24 | 0.23 | **0.72*** | **0.79*** | 1.00 |  |  |  |  |  |  |  |  |  |  |  |  |  |  |  |  |  |  |  |  |  |  |  |
| #16 | 0.30 | | 0.29 | 0.29 | 0.25 | 0.30 | 0.27 | 0.22 | 0.19 | 0.26 | 0.47 | 0.26 | 0.19 | 0.29 | 0.32 | 0.32 | 1.00 |  |  |  |  |  |  |  |  |  |  |  |  |  |  |  |  |  |  |  |  |  |  |
| #17 | 0.15 | | 0.29 | 0.37 | 0.32 | 0.39 | 0.40 | 0.12 | 0.22 | 0.33 | 0.35 | 0.34 | 0.24 | 0.35 | 0.46 | 0.39 | 0.59 | 1.00 |  |  |  |  |  |  |  |  |  |  |  |  |  |  |  |  |  |  |  |  |  |
| **#18^a^** | 0.05 | | 0.28 | 0.38 | 0.35 | 0.45 | 0.46 | 0.21 | 0.32 | 0.33 | 0.37 | 0.22 | 0.33 | **0.70*** | 0.69 | 0.68 | 0.38 | 0.53 | 1.00 |  |  |  |  |  |  |  |  |  |  |  |  |  |  |  |  |  |  |  |  |
| #19 | 0.15 | | 0.27 | 0.45 | 0.48 | 0.52 | 0.55 | 0.22 | 0.31 | 0.44 | 0.36 | 0.37 | 0.32 | 0.70 | 0.68 | 0.66 | 0.33 | 0.44 | **0.71*** | 1.00 |  |  |  |  |  |  |  |  |  |  |  |  |  |  |  |  |  |  |  |
| #20 | 0.20 | | 0.21 | 0.41 | 0.41 | 0.37 | 0.42 | 0.21 | 0.28 | 0.45 | 0.37 | 0.42 | 0.38 | 0.54 | 0.52 | 0.53 | 0.35 | 0.43 | 0.46 | 0.63 | 1.00 |  |  |  |  |  |  |  |  |  |  |  |  |  |  |  |  |  |  |
| #21 | 0.18 | | 0.13 | 0.39 | 0.36 | 0.49 | 0.48 | 0.12 | 0.11 | 0.29 | 0.17 | 0.04 | 0.08 | 0.43 | 0.50 | 0.51 | 0.35 | 0.45 | 0.53 | 0.56 | 0.42 | 1.00 |  |  |  |  |  |  |  |  |  |  |  |  |  |  |  |  |  |
| #22 | 0.23 | | 0.35 | 0.36 | 0.30 | 0.45 | 0.37 | 0.14 | 0.16 | 0.37 | 0.38 | 0.24 | 0.24 | 0.49 | 0.59 | 0.54 | 0.40 | 0.52 | 0.62 | 0.52 | 0.43 | 0.61 | 1.00 |  |  |  |  |  |  |  |  |  |  |  |  |  |  |  |  |
| #23 | 0.07 | | 0.12 | 0.38 | 0.43 | 0.49 | 0.45 | 0.26 | 0.33 | 0.33 | 0.29 | 0.21 | 0.24 | 0.60 | 0.57 | 0.59 | 0.31 | 0.29 | 0.56 | 0.64 | 0.40 | 0.42 | 0.32 | 1.00 |  |  |  |  |  |  |  |  |  |  |  |  |  |  |  |
| #24 | 0.10 | | 0.10 | 0.30 | 0.30 | 0.43 | 0.40 | 0.10 | 0.15 | 0.24 | 0.18 | 0.19 | 0.14 | 0.30 | 0.35 | 0.35 | 0.36 | 0.53 | 0.39 | 0.36 | 0.39 | 0.52 | 0.38 | 0.40 | 1.00 |  |  |  |  |  |  |  |  |  |  |  |  |  |  |
| #25 | 0.34 | | 0.29 | 0.43 | 0.42 | 0.41 | 0.35 | 0.12 | 0.15 | 0.42 | 0.38 | 0.31 | 0.24 | 0.32 | 0.39 | 0.41 | 0.38 | 0.38 | 0.33 | 0.43 | 0.45 | 0.34 | 0.47 | 0.35 | 0.22 | 1.00 |  |  |  |  |  |  |  |  |  |  |  |  |  |
| #26 | 0.31 | | 0.31 | 0.36 | 0.22 | 0.34 | 0.28 | 0.16 | 0.22 | 0.30 | 0.46 | 0.30 | 0.24 | 0.36 | 0.41 | 0.42 | 0.44 | 0.42 | 0.41 | 0.40 | 0.31 | 0.35 | 0.57 | 0.24 | 0.26 | 0.38 | 1.00 |  |  |  |  |  |  |  |  |  |  |  |  |
| #27 | 0.34 | | 0.33 | 0.23 | 0.17 | 0.31 | 0.29 | 0.17 | 0.24 | 0.23 | 0.37 | 0.16 | 0.12 | 0.18 | 0.22 | 0.30 | 0.63 | 0.38 | 0.25 | 0.33 | 0.34 | 0.39 | 0.30 | 0.23 | 0.33 | 0.37 | 0.41 | 1.00 |  |  |  |  |  |  |  |  |  |  |  |
| **#28^a^** | 0.33 | | 0.33 | 0.39 | 0.29 | 0.37 | 0.32 | 0.16 | 0.27 | 0.36 | 0.38 | 0.30 | 0.20 | 0.29 | 0.33 | 0.39 | 0.54 | 0.45 | 0.35 | 0.48 | 0.43 | 0.37 | 0.38 | 0.35 | 0.30 | 0.49 | 0.50 | **0.79*** | 1.00 |  |  |  |  |  |  |  |  |  |  |
| **#29^a^** | 0.05 | 0.17 | | 0.43 | 0.43 | 0.38 | 0.36 | 0.17 | 0.34 | 0.33 | 0.36 | 0.23 | 0.24 | 0.56 | 0.49 | 0.58 | 0.28 | 0.27 | 0.55 | 0.56 | 0.39 | 0.36 | 0.38 | **0.79*** | 0.33 | 0.34 | 0.30 | 0.23 | 0.36 | 1.00 |  |  |  |  |  |  |  |  |  |
| #30 | -0.03 | -0.02 | | 0.23 | 0.19 | 0.26 | 0.27 | 0.11 | 0.20 | 0.08 | 0.22 | 0.15 | 0.09 | 0.30 | 0.29 | 0.31 | 0.18 | 0.08 | 0.32 | 0.36 | 0.19 | 0.20 | 0.20 | 0.40 | 0.23 | 0.15 | 0.22 | 0.12 | 0.22 | 0.44 | 1.00 |  |  |  |  |  |  |  |  |
| #31 | 0.07 | | 0.12 | 0.31 | 0.23 | 0.25 | 0.24 | 0.26 | 0.25 | 0.33 | 0.28 | 0.34 | 0.27 | 0.23 | 0.22 | 0.28 | 0.28 | 0.30 | 0.34 | 0.30 | 0.37 | 0.20 | 0.28 | 0.33 | 0.18 | 0.38 | 0.25 | 0.21 | 0.39 | 0.33 | 0.29 | 1.00 |  |  |  |  |  |  |  |
| #32 | -0.01 | | 0.14 | 0.25 | 0.22 | 0.27 | 0.24 | 0.39 | 0.24 | 0.33 | 0.37 | 0.38 | 0.34 | 0.37 | 0.36 | 0.38 | 0.26 | 0.27 | 0.44 | 0.36 | 0.28 | 0.26 | 0.33 | 0.40 | 0.23 | 0.24 | 0.24 | 0.20 | 0.26 | 0.39 | 0.36 | 0.59 | 1.00 |  |  |  |  |  |  |
| #33 | 0.04 | | 0.13 | 0.10 | 0.12 | 0.21 | 0.20 | 0.18 | 0.22 | 0.25 | 0.27 | 0.18 | 0.18 | 0.12 | 0.13 | 0.02 | 0.27 | 0.22 | 0.19 | 0.20 | 0.15 | 0.17 | 0.18 | 0.14 | 0.34 | 0.09 | 0.17 | 0.25 | 0.20 | 0.19 | 0.53 | 0.11 | 0.28 | 1.00 |  |  |  |  |  |
| #34 | 0.31 | | 0.38 | 0.43 | 0.35 | 0.45 | 0.47 | 0.26 | 0.24 | 0.35 | 0.53 | 0.34 | 0.30 | 0.35 | 0.41 | 0.45 | 0.44 | 0.45 | 0.49 | 0.43 | 0.31 | 0.36 | 0.49 | 0.39 | 0.24 | 0.51 | 0.54 | 0.43 | 0.52 | 0.46 | 0.32 | 0.44 | 0.28 | 0.19 | 1.00 |  |  |  |  |
| #35 | 0.22 | | 0.29 | 0.37 | 0.29 | 0.35 | 0.31 | 0.31 | 0.37 | 0.49 | 0.41 | 0.35 | 0.36 | 0.43 | 0.45 | 0.50 | 0.26 | 0.36 | 0.54 | 0.49 | 0.36 | 0.38 | 0.56 | 0.44 | 0.20 | 0.55 | 0.38 | 0.30 | 0.46 | 0.47 | 0.23 | 0.50 | 0.42 | 0.14 | 0.53 | 1.00 |  |  |  |
| **#36^a^** | 0.34 | | 0.44 | 0.24 | 0.23 | 0.26 | 0.24 | 0.23 | 0.28 | 0.35 | 0.46 | 0.30 | 0.23 | 0.39 | 0.45 | 0.47 | 0.43 | 0.48 | 0.53 | 0.47 | 0.32 | 0.36 | 0.52 | 0.38 | 0.28 | 0.50 | 0.42 | 0.43 | 0.50 | 0.40 | 0.18 | 0.44 | 0.33 | 0.16 | **0.70*** | 0.69 | 1.00 |  |  |
| #37 | 0.11 | | 0.25 | 0.23 | 0.26 | 0.29 | 0.32 | 0.15 | 0.24 | 0.32 | 0.45 | 0.35 | 0.25 | 0.44 | 0.43 | 0.47 | 0.35 | 0.37 | 0.52 | 0.51 | 0.31 | 0.31 | 0.46 | 0.40 | 0.21 | 0.32 | 0.48 | 0.31 | 0.31 | 0.41 | 0.26 | 0.34 | 0.30 | 0.21 | 0.41 | 0.46 | 0.40 | 1.00 |  |
| #38 | 0.13 | | 0.27 | 0.41 | 0.36 | 0.42 | 0.39 | 0.25 | 0.26 | 0.39 | 0.58 | 0.41 | 0.37 | 0.49 | 0.58 | 0.57 | 0.35 | 0.50 | 0.49 | 0.46 | 0.44 | 0.27 | 0.50 | 0.42 | 0.33 | 0.51 | 0.46 | 0.28 | 0.39 | 0.47 | 0.25 | 0.38 | 0.42 | 0.16 | 0.54 | 0.56 | 0.54 | 0.46 | 1.00 |

# Item number in the tentative questionnaire used for this study

a. Items dropped prior to the factor analysis

* Correlation is greater than 0.7, significant at the 0.01 level.
