# Supplementary material for: Development of a scale to assess motivation for competitive employment among persons with severe mental illness
Source: PLoS One. 2018 Oct 2;13(10):e0204809. doi: 10.1371/journal.pone.0204809 (PMC6168136; doi:10.1371/journal.pone.0204809)
Supplement: S5 Table — Frequency of response to each item, including the items which were removed before factor analysis. (DOCX) [file pone.0204809.s005.docx]

S5 Table. Frequency of response to each item of the new scale

|  | 1 | | 2 | | 3 | | 4 | | Mean | SD |
| --- | --- | --- | --- | --- | --- | --- | --- | --- | --- | --- |
| item | n | % | n | % | n | % | n | % |  |  |
| #1 | 5 | 3.7 | 26 | 19.1 | 81 | 59.6 | 24 | 17.6 | 2.9 | 0.7 |
| #2 | 13 | 9.6 | 29 | 21.3 | 72 | 52.9 | 22 | 16.2 | 2.8 | 0.8 |
| #3 | 10 | 7.4 | 28 | 20.6 | 67 | 49.3 | 31 | 22.8 | 2.9 | 0.8 |
| #4 | 7 | 5.1 | 34 | 25 | 67 | 49.3 | 28 | 20.6 | 2.9 | 0.8 |
| #5 | 5 | 3.7 | 29 | 21.3 | 62 | 45.6 | 40 | 29.4 | 3.0 | 0.8 |
| #6 | 5 | 3.7 | 22 | 16.2 | 72 | 52.9 | 37 | 27.2 | 3.0 | 0.8 |
| #7 | 1 | .7 | 7 | 5.1 | 29 | 21.3 | 99 | 72.8 | 3.7 | 0.6 |
| #8 | 1 | .7 | 8 | 5.9 | 47 | 34.6 | 80 | 58.8 | 3.5 | 0.6 |
| #9 | 4 | 2.9 | 9 | 6.6 | 58 | 42.6 | 65 | 47.8 | 3.4 | 0.7 |
| #10 | 2 | 1.5 | 18 | 13.2 | 64 | 47.1 | 52 | 38.2 | 3.2 | 0.7 |
| #11 | 2 | 1.5 | 11 | 8.1 | 66 | 48.5 | 57 | 41.9 | 3.3 | 0.7 |
| #12 | 1 | .7 | 6 | 4.4 | 47 | 34.6 | 82 | 60.3 | 3.5 | 0.6 |
| #13 | 8 | 5.9 | 19 | 14 | 51 | 37.5 | 58 | 42.6 | 3.2 | 0.9 |
| #14 | 6 | 4.4 | 24 | 17.6 | 60 | 44.1 | 46 | 33.8 | 3.1 | 0.8 |
| #15 | 7 | 5.1 | 27 | 19.9 | 58 | 42.6 | 44 | 32.4 | 3.0 | 0.9 |
| #16 | 6 | 4.4 | 22 | 16.2 | 56 | 41.2 | 52 | 38.2 | 3.1 | 0.8 |
| #17 | 8 | 5.9 | 31 | 22.8 | 61 | 44.9 | 36 | 26.5 | 2.9 | 0.9 |
| #18 | 6 | 4.4 | 25 | 18.4 | 60 | 44.1 | 45 | 33.1 | 3.1 | 0.8 |
| #19 | 5 | 3.7 | 18 | 13.2 | 57 | 41.9 | 56 | 41.2 | 3.2 | 0.8 |
| #20 | 7 | 5.1 | 22 | 16.2 | 49 | 36 | 58 | 42.6 | 3.2 | 0.9 |
| #21 | 8 | 5.9 | 33 | 24.3 | 74 | 54.4 | 21 | 15.4 | 2.8 | 0.8 |
| #22 | 8 | 5.9 | 32 | 23.5 | 70 | 51.5 | 26 | 19.1 | 2.8 | 0.8 |
| #23 | 8 | 5.9 | 19 | 14 | 58 | 42.6 | 51 | 37.5 | 3.1 | 0.9 |
| #24 | 17 | 12.5 | 51 | 37.5 | 51 | 37.5 | 17 | 12.5 | 2.5 | 0.9 |
| #25 | 5 | 3.7 | 17 | 12.5 | 77 | 56.6 | 37 | 27.2 | 3.1 | 0.7 |
| #26 | 23 | 16.9 | 55 | 40.4 | 43 | 31.6 | 15 | 11 | 2.4 | 0.9 |
| #27 | 5 | 3.7 | 14 | 10.3 | 68 | 50 | 49 | 36 | 3.2 | 0.8 |
| #28 | 3 | 2.2 | 13 | 9.6 | 68 | 50 | 52 | 38.2 | 3.2 | 0.7 |
| #29 | 6 | 4.4 | 18 | 13.2 | 56 | 41.2 | 56 | 41.2 | 3.2 | 0.8 |
| #30 | 18 | 13.2 | 36 | 26.5 | 38 | 27.9 | 44 | 32.4 | 2.8 | 1.0 |
| #31 | 4 | 2.9 | 15 | 11 | 53 | 39 | 64 | 47 | 3.3 | 0.8 |
| #32 | 3 | 2.2 | 17 | 12.5 | 49 | 36 | 67 | 49.3 | 3.3 | 0.8 |
| #33 | 30 | 22.1 | 40 | 29.4 | 39 | 28.7 | 27 | 19.9 | 2.5 | 1.0 |
| #34 | 5 | 3.7 | 22 | 16.2 | 64 | 47.1 | 45 | 33.1 | 3.1 | 0.8 |
| #35 | 4 | 2.9 | 11 | 8.1 | 54 | 39.7 | 67 | 49.3 | 3.4 | 0.8 |
| #36 | 4 | 2.9 | 15 | 11 | 60 | 44.1 | 57 | 41.9 | 3.3 | 0.8 |
| #37 | 20 | 14.7 | 42 | 30.9 | 45 | 33.1 | 29 | 21.3 | 2.6 | 1.0 |
| #38 | 4 | 2.9 | 17 | 12.5 | 64 | 47.1 | 51 | 37.5 | 3.2 | 0.8 |
| overall | 10 | 7.4 | 38 | 27.9 | 54 | 39.7 | 34 | 25 | 2.8 | 0.9 |

# Item number in the tentative questionnaire used for this study
