# Supplementary material for: Development of a scale to assess motivation for competitive employment among persons with severe mental illness
Source: PLoS One. 2018 Oct 2;13(10):e0204809. doi: 10.1371/journal.pone.0204809 (PMC6168136; doi:10.1371/journal.pone.0204809)
Supplement: S1 Fig — (DOCX) [file pone.0204809.s006.docx]

S1 Fig. Process of creating the new measure

Create an item pool

- Review of previous research

- Focus group interview

Feedback from researchers and three peer workers

A 108-item pool

A tentative draft consisting of 39 items

Item reduction based on the KJ method

An exploratory factor analysis

A provisional scale was completed by 136 respondents

A provisional scale consisting of 38 items

An item (“I want to pursue my career goals”) was removed from the draft due to its ambiguity

A four-factor scale with 23 items

Removal of seven items (#3, #6, #13, #18, #28, #29, #36)

which highly correlated with other items
